# Silent Burden of Mercury: Environmental Exposure and Determinants Among Indigenous Communities in the Colombian Amazon

**DOI:** 10.3390/toxics14100880

**Published:** 2026-09-30

**Authors:** Roberth de Jesús Paternina-Uribe, Carlos Federico Molina-Castaño, Catalina María Arango-Alzate, María Luisa Toro-Oviedo, Javier Alonso Ruíz-Guzmán, Angelly Paternina-Vergara, Clelia Calao-Ramos, Santiago Español Cano, Ruth Marien Palma Parra, Sonia Mireya Díaz Criollo, José Luis Marrugo-Negrete

**Affiliations:** 1 Research Group on Water, Applied Chemistry, and Environmental Science, University of Córdoba, Montería 230002, Colombia; mariatoro@correo.unicordoba.edu.co (M.L.T.-O.); javierruizg@correo.unicordoba.edu.co (J.A.R.-G.); angellypaterninav@gmail.com (A.P.-V.); cleliacalaor@correo.unicordoba.edu.co (C.C.-R.); jmarrugo@correo.unicordoba.edu.co (J.L.M.-N.); 2 BISMA Research Group, Tecnológico de Antioquia, Medellín 050034, Colombia; cmolina@tdea.edu.co; 3 Biociencias Research Group, Faculty of Health Sciences, Colegio Mayor de Antioquia, Medellín 050034, Colombia; nucatara@gmail.com; 4 Fundación Almadén “Francisco Javier de Villegas”, Almadén, 13400 Ciudad Real, Spain; sant_esp@hotmail.com; 5 Instituto Nacional de Salud, Ministerio de Salud y Protección Social, Bogotá 111321, Colombia; rpalma@ins.gov.co (R.M.P.P.); sonia.diaz2118@gmail.com (S.M.D.C.)

**Keywords:** mercury, exposure, indigenous communities, Amazon, biomonitoring, methylmercury, fish consumption, Colombia

## Abstract

**Highlights:**

**What are the main findings?**

- Indigenous communities in the Colombian Amazon show mercury levels far exceeding international safety thresholds in blood, urine, and hair, despite no direct mining involvement.
- Fish consumption from the Apaporis River was the strongest dietary predictor of elevated mercury concentrations.

**What are the implications of the main findings?**

- Mercury contamination in Amazonian watersheds is a transboundary issue requiring coordinated biomonitoring and policy intervention.
- Targeted dietary guidance and environmental health programs are urgently needed to protect vulnerable indigenous populations.

**Abstract:**

Mercury contamination remains one of the most persistent environmental and public health challenges in the Amazon, where fish consumption represents the primary pathway of exposure to methylmercury. This study assessed the prevalence and determinants of mercury exposure among indigenous communities of Taraira, Vaupés, in the Colombian Amazon. A cross-sectional survey was conducted in 2017 across eleven communities affiliated with the Association of Indigenous Captains of Taraira (ACITAVA). Sociodemographic, occupational, and dietary data were collected through structured interviews, and total mercury concentrations were determined in blood, urine, and hair samples using thermal decomposition–amalgamation atomic absorption spectrophotometry (EPA 7473). Among 238 participants (56.3% male; mean age 36.9 years), mean mercury concentrations were 80.4 µg/L in blood, 15.1 µg/L in urine, and 19.8 µg/g in hair, with 100% of blood and hair samples exceeding international reference limits. Higher mercury levels were significantly associated with fish consumption from the Apaporis River, residence in multiple locations within the municipality, and secondary education level (*p* < 0.05). In multivariate analysis, blood mercury was the strongest predictor of hair mercury (β = 0.707, *p* < 0.001), and the model explained 64.5% of the variance. These findings reveal chronic environmental mercury exposure among indigenous populations not directly involved in mining activities, emphasizing the transboundary nature of contamination in Amazonian watersheds. Strengthening biomonitoring programs and promoting safer dietary practices are essential to protect the health and cultural integrity of these vulnerable communities.

## 1. Introduction

Heavy metal contamination remains one of the most pressing global environmental and public health challenges due to its persistence, bioaccumulative nature, and toxicity [1]. Unlike organic pollutants, heavy metals are not biodegradable, so they persist in environmental compartments and bioaccumulate in organisms, progressively biomagnifying along the food chain [2]. Among these contaminants, mercury (Hg) stands out for its high toxicity and ability to transform into methylmercury (MeHg), a highly neurotoxic organic form that biomagnifies through aquatic ecosystems, posing a major threat to populations that rely on fish as their primary source of protein [3,4,5,6].

Mercury is a potent neurotoxic element that poses a serious threat to human health and the environment. Its toxic effects have been extensively documented over the past decades, affecting multiple organ systems, particularly the central nervous system [7,8,9]. Both inorganic and organic forms of mercury, especially methylmercury, can disrupt neuronal development, impair cognitive and motor functions, and produce long-term neurological and reproductive consequences even at low levels of exposure [7,8,9]. Numerous epidemiological and toxicological investigations have confirmed its neurotoxic, immunotoxic, and teratogenic effects in occupationally exposed populations as well as in communities living near contaminated aquatic ecosystems [9,10,11,12,13]. Owing to its persistence, global distribution, and bioaccumulative nature, mercury is now recognized as one of the most critical environmental pollutants of worldwide concern [14].

In Latin America, artisanal and small-scale gold mining (ASGM) has emerged as the leading source of atmospheric and aquatic mercury emissions. It is estimated that this activity releases between 800 and 1000 metric tons of mercury annually, with approximately half originating from Latin American countries [15]. In Colombia, informal and illegal gold extraction is particularly widespread in regions such as Chocó, Antioquia, Bolívar, and the Amazon basin, where metallic mercury is commonly used for gold amalgamation. The volatilization and inadequate disposal of this element have resulted in severe contamination of air, soil, and water, as well as high mercury concentrations in fish consumed by local populations [16,17,18,19,20,21].

Indigenous populations inhabiting the Amazonian river basins represent one of the most vulnerable groups to mercury exposure. Their deep dependence on aquatic ecosystems and fish-based diets, combined with their proximity to mining-impacted watersheds, creates an elevated risk of methylmercury bioaccumulation. Previous studies conducted in Amazonian communities of Brazil and Colombia have reported mercury levels exceeding international safety thresholds in both blood and hair, even among individuals with no direct occupational exposure, suggesting secondary contamination through interconnected river networks [3,12,22,23,24].

Within this context, the Vaupés department in the Colombian Amazon is home to several indigenous communities that, despite the absence of local mining activity, may experience indirect mercury exposure through the consumption of contaminated fish. These communities inhabit the Apaporis River basin, which is hydrologically connected to gold-mining areas such as the Taraira and Caquetá rivers, enabling long-distance contaminant transport across the watershed. However, data on the magnitude and determinants of mercury exposure in this region remain extremely limited.

Therefore, the present study aimed to assess the prevalence and associated factors of mercury exposure among indigenous communities in Taraira, Vaupés (Colombian Amazon), by quantifying total mercury concentrations in biological matrices (blood, urine, and hair) and analyzing socio-demographic, environmental, and dietary variables. By generating empirical evidence from a scarcely studied population, this research contributes to a better understanding of chronic mercury exposure dynamics in Amazonian ecosystems and provides essential data for environmental health surveillance and public policy interventions in vulnerable indigenous settings.

## 2. Materials and Methods

### 2.1. Study Design and Population

**Figure 1:**
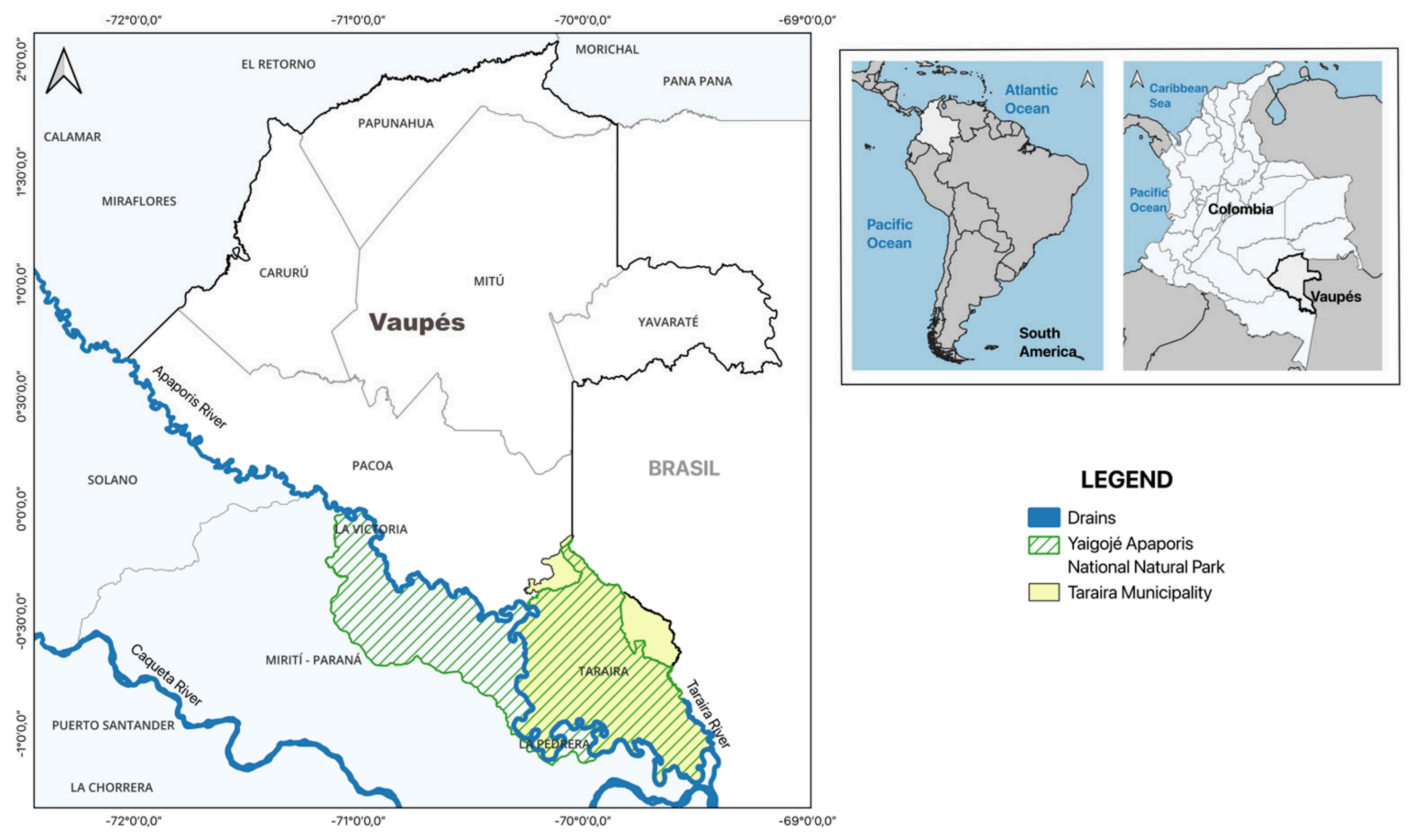
Location map of the municipality of Taraira, Department of Vaupés, Colombia. Insets show the position of Colombia within South America and of the Vaupés department within Colombia. The Yaigojé Apaporis National Natural Park and the Apaporis, Caquetá, and Taraira rivers are indicated.

A descriptive cross-sectional study was conducted between July and September 2017 in eleven indigenous communities affiliated with the Asociación de Capitanes Indígenas de Taraira Vaupés (ACITAVA), located in Yaigojé Apaporis National Natural Park, the municipality of Taraira, in southeastern Colombia (Figure 1). These communities, situated within the Amazon rainforest, are characterized by geographical isolation, limited access to health services, and subsistence economies heavily dependent on fishing and small-scale agriculture. Although no gold-mining activity occurs within their territories, their proximity to the Taraira and Caquetá river basins, areas of intense artisanal mining, could place them at potential environmental risk of mercury exposure through hydrological connectivity and fish consumption.

The target population comprised men and women aged 18 years or older who had lived continuously in the study area for at least six months.

### 2.2. Sample Size and Selection

The sample size was calculated using a finite population correction, based on 355 registered adults across the eleven communities, according to a census conducted in each community by its leader. Assuming a 50% expected prevalence of mercury exposure, a 95% confidence level, and a 5% margin of error, the required sample size was 185 participants; however, after completing the fieldwork and laboratory analysis, 238 individuals with complete samples were obtained. A simple random sampling approach was used, with proportional representation from each community. Recruitment and logistics were coordinated with local leaders to ensure transparency and adherence to cultural protocols.

### 2.3. Fieldwork and Data Collection

Fieldwork was carried out jointly by professionals from the National Institute of Health, the University of Córdoba, and trained field staff, including physicians and nurses, with the support of local Indigenous guides and translators. Prior to sampling, meetings were held with community captains and indigenous authorities to explain the study’s objectives and procedures.

Participants completed a structured questionnaire adapted from the health-assessment instrument of the Guidance for identifying populations at risk from Hg exposure (UNEP/WHO) [25], designed to capture socio-demographic information, lifestyle habits, occupational and environmental exposure histories, and fish consumption patterns. All study participants had their body weight measured using a Kenwell BR2016 mechanical beam scale (Camry Electronic, Zhaoqing, China). To estimate the amount of fish consumed at each meal, several groups of participants were presented with a container (bowl) containing various fish species they reported eating. They were asked to indicate the most common size of fish they consumed or how many portions they divided larger fish into. These portions were then weighed, resulting in an average portion size of 364 g. This information, along with the frequency of consumption reported in the surveys, was used to estimate weekly fish intake. Each participant’s weekly intake of mercury and methylmercury was calculated using the formula established by UNEP/WHO [25], multiplying the weekly fish intake (kg) by the average mercury concentration in the fish consumed (µg/kg), and then dividing by the person’s body weight (kg).

### 2.4. Biological Sampling and Mercury Analysis

Biological samples of hair, blood, and urine were collected from all participants, as well as samples of the different fish species they reported consuming most frequently. Hair samples were cut from the occipital region, close to the scalp, using stainless steel scissors (Super—Dent Ltd., Bogotá, Colombia, manufactured in Pakistan) and stored in labeled paper envelopes [26]. Venous blood samples were drawn into EDTA-coated Vacutainer tubes [27], while urine samples were collected in sterile polypropylene containers after providing participants with detailed hygiene instructions [28]. Fish samples were taken from the dorsal muscle using surgical scalpels and stored in labeled plastic bags. All samples were kept at 4 °C and transported to the National Institute of Health for analysis in insulated 22 L plastic cool boxes with ice and a built-in thermometer (ESTRA STR-22 L, Medellín, Colombia), thus maintaining the cold chain.

Total mercury concentrations for hair, blood and fish were determined using Thermal Decomposition Amalgamation Atomic Absorption Spectrophotometry according to EPA Method 7473 [29], employing a TriCell Milestone DMA-80 Direct Mercury Analyzer (Milestone S.r.l., Bergamo, Italy). Prior to analysis, blood samples were homogenized by vortexing; 100 µL of each sample was measured using a transfer pipette and deposited into quartz cells. Hair samples were washed with distilled water and acetone to remove interfering material, then dried and finely cut; 200 mg portions were weighed into nickel cells. Fish samples were lyophilized at −50 °C and 0.13 mbar, using a Labconco Free Zone 2.5 freeze-dryer (Labconco Corporation, Kansas City, MO, USA), homogenized by mortar-and-pestle grinding, and subsequently weighed (15–30 mg) for analysis. Total mercury concentrations in urine samples were determined by atomic absorption spectrometry with Zeeman correction—based on EPA method 7473, using a LUMEX RA-915M Direct Mercury Analyzer (Lumex Instruments, Mission, BC, Canada) coupled to a PYRO-915+ pyrolyzer furnace (Lumex Instruments, Mission, BC, Canada). Methylmercury concentrations in hair and fish samples were also quantified using the Milestone DMA-80 TriCell direct mercury analyzer, in accordance with method EUR-25830EN-2013 of the European Commission Joint Research Centre, following alkaline extraction of the hair samples (50 mg of sample with 10 M NaOH and 1% L-cysteine in a 2:1 ratio, at 90 °C in a water bath for one hour) and acid extraction of the fish samples (0.5 g of sample with 5.0 mL of HBr and 8.0 mL of toluene added), and mixing of the extracts with L-cysteine. The detection limits of the method, determined as three times the standard deviation of 10 blank samples, were 0.0025 µg/g for hair and fish; 0.015 µg/g and 0.003 µg/g for methylmercury in hair and fish, respectively; 2.6 µg/L for blood and 0.34 µg/L for urine. The accuracy of the methods was determined with reference materials (CRM-397, IAEA-086 and DORM-2) with a recovery between 96.0 ± 2.2% and 98.7%. In this study, the reference values for mercury in blood and urine were those derived by the German Human Biomonitoring Commission of the German Environment Agency (UBA), in this case HBM-I, as the values above which health risks associated with mercury exposure cannot be ruled out; these values have also been adopted and recommended as reference values by the WHO (Reference value: blood = 5 µg/L, urine = 7 µg/L) [25,30]. For mercury in hair, the reference value suggested by the US EPA (1 µg/g) was used [31].

### 2.5. Statistical Analysis

Data were entered into a Microsoft Excel spreadsheet, in which range and consistency checks were applied to validate the records, and were then exported to IBM SPSS Statistics version 22.0 for analysis. Descriptive statistics were used to summarize socio-demographic and exposure variables, expressed as means and standard deviations for continuous variables and frequencies for categorical variables. Normality was assessed using the Shapiro–Wilk test. Differences in mean mercury concentrations between groups were evaluated using *t*-tests or ANOVA for normally distributed variables and the Mann–Whitney U or Kruskal–Wallis tests for non-parametric data. Categorical associations were tested using the chi-square (χ^2^) test.

Correlations between mercury concentrations in hair, blood, and urine and continuous covariates were assessed using Spearman’s rank correlation coefficient. A multiple linear regression model was constructed to identify factors independently associated with hair mercury concentrations. Candidate variables for the multivariable analysis were selected using a combination of epidemiological relevance, biological plausibility, and statistical evidence. Variables showing an association with the outcome at *p* < 0.25 in the bivariate analysis were considered for inclusion, together with variables regarded a priori as potential confounders based on previous epidemiological and toxicological knowledge, irrespective of their bivariate statistical significance.

Model building was performed sequentially, evaluating the contribution of candidate variables and potential confounding effects. Variables were retained according to their epidemiological relevance, biological plausibility, statistical contribution to the model, and changes in the estimates of the main exposure variables. Competing models were compared using likelihood-based criteria and the coefficient of determination (R^2^).

Potential multicollinearity among explanatory variables was assessed using tolerance and the variance inflation factor (VIF). Model assumptions were evaluated by examining linearity between continuous predictors and the outcome, normality and homoscedasticity of residuals, independence of errors, and the presence of influential observations. Statistical significance in the final multivariable model was established at *p* < 0.05.

### 2.6. Ethical Considerations

The study was conducted in accordance with national and international ethical standards for research involving human participants. Initially, indigenous authorities granted their verbal authorization through community assemblies, in accordance with customs, intercultural ethical principles, and respect for autonomy. Subsequently, all individuals voluntarily agreed to participate after receiving clear and comprehensive information about the study objectives and procedures. Written informed consent was obtained from each participant prior to the collection of biological samples and survey data. The research protocol and consent forms were reviewed and approved by the Institutional Ethics Committee of the National Institute of Health of Colombia (Minutes No. FOR-R03.0000-05 of 19 July 2012; Project Code 36-2015). Individual results were confidentially communicated to participants and local health representatives, while collective findings were presented to community leaders to support environmental and public health surveillance initiatives.

## 3. Results

The sociodemographic and nutritional characteristics of participants are summarized in Table 1. A total of 238 individuals from eleven indigenous communities in the municipality of Taraira, Vaupés, participated in the study. Of these, 56.3% (*n* = 134) were men and 43.7% (*n* = 104) were women. Most participants were married or in stable unions (76.5%), and educational attainment was generally low (14.8% were illiterate and 50.4% had completed primary school). Regarding residential history, 24.5% had lived in the same area since birth, 22.3% in another area within the same municipality, and 48.9% in a different department. The main occupation was agriculture (70.1%). Lifestyle characteristics showed that 58.8% had a history of smoking and 73.5% reported alcohol consumption. Regarding fish consumption, the majority (70%) obtained fish from areas where mining did not take place, with most indicating that they sourced them from the Apaporis River (Table 1).

Table 2 outlines the mercury and methylmercury concentrations in the sampled fish species, which correspond to the species most frequently consumed by the study participants. Mercury concentrations across all species ranged from 8.4 µg/kg to 2524.7 µg/kg (mean: 542.8 ± 576.1 µg/kg) and methylmercury concentrations ranged from 6.6 µg/kg to 2411.7 µg/kg (mean: 485.2 ± 516.8 µg/kg); the proportion of total mercury present as methylmercury was 87.8 ± 5.4%. Most of the species were carnivorous, and these exhibited the highest mean mercury and methylmercury concentrations compared with the other sampled species, which were omnivorous.

Other sociodemographic variables, the estimated weekly mercury intake, and mercury concentrations in human matrices are presented in Table 3. All blood and hair samples, and 79.8% of urine samples, exceeded the international reference limits described in the Section 2.

Significant associations with hair mercury concentrations were observed for education level, residential history, and fish source. Participants with secondary education had higher mean hair mercury levels (21.8 ± 12.2 µg/g) than illiterate individuals (15.9 ± 7.2 µg/g; *p* = 0.023). Those who had lived in different areas of the same municipality exhibited higher concentrations (24.5 ± 13.6 µg/g) than those who had never moved (14.9 ± 5.2 µg/g; *p* < 0.001). Individuals who obtained fish from the Apaporis River had higher hair mercury concentrations (21.5 ± 10.6 µg/g) than those consuming fish from both the Apaporis River and its tributary streams (17.7 ± 9.7 µg/g) or other rivers (16.0 ± 8.1 µg/g; *p* = 0.001). No other variables showed significant associations (Table 4).

Spearman’s correlation analysis revealed a positive correlation between age and hair mercury levels and a negative correlation with residence time. Hair mercury concentrations were strongly correlated with hair methylmercury, blood mercury and moderately correlated with urinary mercury (Table 5).

The multivariate linear regression model identified five predictors of hair mercury concentrations. Women showed slightly lower mercury levels in hair than men. Participants consuming fish from streams or rivers other than the Apaporis presented lower concentrations than those obtaining fish from the Apaporis River. Age was inversely associated with hair mercury. For each 1 µg/L increase in blood mercury, hair mercury increased by 0.707 µg/g, and for each 1 µg/L increase in urinary mercury, it increased by 0.102 µg/g. The model explained 64.5% of the variability in hair mercury levels (R^2^ = 0.645) (Table 6).

Notably, the direction of the association between age and hair mercury changed after multivariable adjustment. Although age showed a weak positive crude correlation with hair mercury in the Spearman analysis (ρ = 0.128, *p* = 0.048), its adjusted regression coefficient was negative (β = −0.120, *p* = 0.008). This reversal suggests that the crude age–mercury relationship was influenced by the distribution of other covariates included in the model.

## 4. Discussion

The high average concentrations of mercury found in this study in blood (80.4 µg/L), urine (15.1 µg/L) and hair (19.8 µg/g), methylmercury in hair (16.8 µg/g) and the high average weekly intake of methylmercury through fish consumption (29.0 µg/kg/week), which far exceed the reference limits, revealed a widespread and elevated exposure to mercury among indigenous communities in the municipality of Taraira, Vaupés.

The German Human Biomonitoring (HBM) Commission of the German Environment Agency (UBA) has established that, in exposure situations where blood mercury concentrations exceed 15 µg/L, immediate intervention is required to halt exposure and obtain medical attention to assess potential signs of adverse health effects [30]; likewise, the UNEP/WHO guidance document on identifying populations at risk from mercury exposure establishes that regulatory measures must be taken to prevent potential health harm in populations where the average hair mercury concentration exceeds 2 µg/g and mercury weekly intake surpasses the provisional tolerable intake (1.6 µg/kg/week for sensitive populations and 3.2 µg/kg/week for the general population) [25].

In the present study, 92.4% of blood samples had concentrations greater than 40 µg/L, and 93.3% and 84.0% of hair samples had concentrations greater than 10 µg/g of mercury and methylmercury, respectively. Furthermore, more than 95% of participants reported a weekly intake of mercury and methylmercury greater than 3.2 µg/kg/week. These findings provide compelling evidence of chronic environmental exposure in populations without direct involvement in mining activities, highlighting the extensive transport and persistence of mercury across Amazonian ecosystems.

The high average concentration of methylmercury in hair (17.2 µg/g) and the average methylmercury weekly intake (45.3 µg/kg/week) among pregnant women are particularly concerning. The hair levels exceed the methylmercury concentrations reported in the hair of mothers exposed during pregnancy through fish consumption in some of the most significant global studies, such as those in the Faroe Islands (geometric mean: 4.3 µg/g) and New Zealand (mean: 8.3 µg/g), which were associated with adverse effects on the neurological development of the children [32]. Furthermore, the methylmercury weekly intake exceeded by more than tenfold the value considered safe (1.6 µg/kg/week) for the protection of developing fetuses [25].

A previous study conducted two years earlier in four of the 11 indigenous communities included in the present study reported similar hair mercury concentrations (mean: 22.98 ± 1.19 µg/g; range: 2.6–63.7 µg/g) [19], indicating a scenario of sustained exposure over time. The current results are also consistent with findings from other studies conducted in the Tapajós River basin in Brazil, where riverine communities dependent on fish exhibited similar levels [9,33,34,35,36]. In the current study, the predominant exposure pathway was fish consumption, particularly carnivorous and omnivorous species caught in the Apaporis River. This dietary pattern, typical of Amazonian subsistence populations, favors bioaccumulation of methylmercury, the organic and neurotoxic form that easily crosses the blood–brain and placental barriers [9,33,34,35,36].

The observation that participants consuming fish from the Apaporis River had the highest mercury concentrations supports the hypothesis that interconnected river basins act as secondary contamination sources. Similar findings have been reported in studies from the Madeira and Tapajós basins, where mercury released from upstream gold-mining activities is transported downstream and biomagnified throughout the aquatic food web [13,23,35]. These data underscore that mercury pollution in the Amazon should be regarded as a transboundary and watershed-scale issue rather than a localized phenomenon.

The strong correlation observed between hair and blood mercury concentrations (ρ = 0.785, *p* < 0.001) indicates internal consistency in exposure assessment and confirms hair as a reliable biomarker of chronic methylmercury exposure, as demonstrated in previous Amazonian studies [9,24,35,37]. Urinary mercury concentrations were lower relative to their reference value than were blood and hair concentrations, and they correlated only moderately with hair mercury. Several aspects can be considered in the interpretation: the results showed a population with high exposure due to the consumption of fish containing mostly methylmercury (Table 2); however, a small fraction may be inorganic mercury, which is mostly excreted in the urine. It is well known that only a fraction of methylmercury (around 10%) is excreted in the urine, after its demethylation in internal tissues [38,39,40]; therefore, these small excreted fractions can increase concentrations in urine, while increasing to a greater extent in blood and hair, when fish consumption is high and sustained over time, as occurs in the indigenous communities evaluated in this study, which could explain the correlation between concentrations in hair and urine. On the other hand, this correlation could suggest the coexistence of organic and inorganic mercury exposure pathways, due to the combination of food intake and possible environmental contact with contaminated sediments, water or air [41,42,43], taking into account the gold mining processes that are developed in the neighboring basins of the Taraira and Caquetá rivers, where mercury contamination has been described in water, sediment and soil of the jungle [44]; however, more detailed and follow-up studies would be necessary to verify these assumptions.

Sociodemographic characteristics also influenced mercury levels. Participants who had lived in multiple locations within the municipality presented significantly higher hair mercury concentrations than those who had never relocated, possibly reflecting differential access to fishing sites or exposure to more contaminated tributaries. This finding aligns with earlier research documenting the role of mobility and land use patterns in shaping exposure profiles among Amazonian populations [45,46]. Furthermore, individuals with higher education levels exhibited greater mercury levels, which may be related to dietary diversity or a preference for larger predatory fish, species known to contain higher methylmercury burdens [47,48]. An apparently contradictory pattern was observed for age. In the bivariate analysis, age showed a weak positive correlation with hair mercury concentrations (Spearman’s ρ = 0.128, *p* = 0.048), whereas in the multivariable model its adjusted association became negative (β = −0.120, *p* = 0.008). This change in direction is consistent with negative or qualitative confounding, and potentially with a suppression effect. Spearman’s correlation represents the crude relationship between age and hair mercury without accounting for other determinants of exposure, whereas the regression coefficient estimates the association with age conditional on the other covariates included in the model, particularly biological mercury markers and fish-source variables. Therefore, the positive crude association may reflect differences in exposure patterns across age groups that were accounted for after multivariable adjustment. Once these factors were controlled, the independent association between age and hair mercury became inverse. Given the cross-sectional nature of the study, however, this finding should be interpreted as an adjusted association rather than evidence of a causal age effect.

Although mercury concentrations were elevated in both sexes, women showed a tendency toward lower hair mercury concentrations compared with men. However, this association was not statistically significant after multivariable adjustment (β = −0.063, *p* = 0.166). Therefore, the observed pattern should be interpreted cautiously and does not provide evidence of an independent association between sex and hair mercury concentrations in this population. Lower hair mercury concentrations in women have nevertheless been reported in other populations, where they have been attributed to variations in body composition, hormonal influences on metal metabolism, and possible differences in dietary habits [24,40]. The present data do not allow these mechanisms to be evaluated.

The inverse correlation between length of residence and mercury levels could reflect gradual changes in diet or adaptive behaviors that reduce exposure over time, although a longitudinal design would be necessary to confirm this hypothesis. In contrast, the regression analysis demonstrated that blood mercury was the strongest predictor of hair mercury concentration, reinforcing the biological coherence of these exposure indicators and their utility in biomonitoring studies.

A major strength of this study lies in the simultaneous assessment of mercury concentrations in three biological matrices: hair, blood, and urine, allowing for a comprehensive evaluation of both recent and chronic exposure. The use of standardized sampling and analytical protocols, coupled with analysis in certified laboratories, enhances the reliability and reproducibility of the findings. Another key strength was the participatory approach involving indigenous leaders and communities, which facilitated cultural acceptability and trust, ensuring accurate data collection and informed consent processes. Nevertheless, certain limitations should be acknowledged: although gold mining in the Colombian Amazon has been increasing [49], the present study was conducted nine years ago, so the current situation may be different; the population evaluated corresponds to indigenous communities in a specific area (National Natural Park), therefore the results cannot be generalized to other Amazonian regions with different ecological or socioeconomic conditions; and the data based on self-reporting may have introduced recall bias.

## 5. Conclusions

The results showed that, for the general population evaluated, including pregnant women, average values for blood mercury concentration, hair methylmercury concentration, and weekly methylmercury intake exceeded the reference values considered safe for health by more than ten times.

Based on the above, this study demonstrates a high level of environmental mercury exposure among indigenous communities in the Colombian Amazon, primarily associated with the consumption of contaminated fish from river systems hydrologically connected to mining zones. The findings confirm mercury’s long-range environmental persistence and highlight the disproportionate vulnerability of populations that depend directly on aquatic ecosystems for food and livelihood.

Public health authorities should strengthen environmental and biomonitoring programs in Amazonian territories, prioritizing indigenous populations and women of reproductive age. Intersectoral policies are urgently needed to regulate artisanal gold-mining practices in shared watersheds and to promote sustainable alternatives for local economies. In parallel, community-based health education programs should emphasize the risks of consuming contaminated fish and encourage dietary diversification toward species with lower trophic levels and reduced bioaccumulation potential.

Ultimately, this research contributes critical evidence for designing targeted interventions that integrate environmental, health, and cultural dimensions. Establishing coordinated efforts between environmental agencies, health institutions, and indigenous organizations will be essential to mitigate mercury exposure and safeguard both human and ecosystem health in the Amazon basin.

## Figures and Tables

**Table 1:** Sociodemographic, health, and nutritional characteristics of the indigenous communities in Vaupés.

Variable	Category	Frequency	%
Sex	Male	134	56.3
	Female	104	43.7
Marital status	Married/Stable union	179	76.5
	Single/Separated/Widowed	55	23.5
Education	Illiterate	35	14.8
	Primary	119	50.4
	Secondary	68	28.8
	Higher	14	5.9
Residential history	Same area since birth	57	24.5
	Other area within same municipality	52	22.3
	Other municipality of the same department	10	4.3
	Different department	114	48.9
Occupation	Farmer	162	70.1
	Fisher	14	6.1
	Other trades/Admin	55	23.8
Have you ever smoked	Yes	140	58.8
	No	98	41.2
Current smoking	Yes	123	51.9
	No	114	48.1
Alcohol consumption	Yes	175	73.5
	No	63	26.5
Days per week with fish consumption	2	2	0.8
	3	7	3.0
	4	7	3.0
	5	17	7.1
	6	24	10.1
	7	181	76.0
Fish sourcing area	Non-mining areas	166	70.0
	Mining areas	62	26.2
	Unknown	9	3.8
Fish capture site	Apaporis River	132	56.4
	Apaporis River and its tributaries	85	36.3
	Other rivers	17	7.3

**Table 2:** Total mercury (Hg) and methylmercury (MeHg) concentrations in the sampled fish species.

Fish (Scientific Name)	FH	*n*	Hg (µg/kg)		MeHg (µg/kg)		% MeHg	Total Length (cm)	
			Min–Max	Mean ± SD	Min–Max	Mean ± SD		Min–Max	Mean ± SD
Apuy (*Brachyplatystoma juruense*)	C	17	517.4–2280.2	1019.6 ± 442.4	460.5–2183.7	958.4 ± 428.6	93.5 ± 2.7	34.6–64.0	49.7 ± 9.9
Bagre-Barbudo (*Leiarus marmoratus*)	O	4	167.1–222.3	197.3 ± 22.9	134.6–187.2	165.3 ± 24.6	83.6 ± 4.6	58.5–62.3	60.2 ± 1.6
Barbachato (*Pinirampus pinirampu*)	C	9	757.9–1672.3	1284.1 ± 334.2	689.7–1614.4	1207.0 ± 321.9	93.9 ± 2.5	34.6–60.8	47.6 ± 7.9
Bocón o Jura Jura (*Ageneiosus inermis*)	C	11	382.4–2524.7	1194.4 ± 697.4	341.2–2411.7	1106.7 ± 657.9	92.0 ± 3.3	38.7–65.3	54.4 ± 8.6
Caloche (*Sternopygus macrurus*)	C	8	115.5–466.6	259.1 ± 118.2	93.7–432.1	224.2 ± 110.6	85.4 ± 4.0	17.0–31.3	24.6 ± 5.3
Dormilón o Moncholo (*Hoplias malabaricus*)	C	12	217.6–389.3	309.5 ± 63.0	181.2–367.4	271.5 ± 63.2	87.4 ± 6.8	18.0–28.5	24.3 ± 3.5
Guabina (*Rhamdia* sp.)	C	14	83.9–268.0	149.1 ± 54.4	70.1–218.5	128.7 ± 47.0	86.2 ± 3.4	27.0–56.0	39.2 ± 8.4
Omima o Guaracú (*Leporinus friderici*)	O	14	31.6–95.3	60.1 ± 16.3	27.1–82.9	52.0 ± 13.9	86.6 ± 2.6	20.0–38.6	28.4 ± 6.6
Palometa (*Metynnis hypsauchen*)	O	23	8.4–129.9	44.4 ± 27.9	6.6–118.5	37.6 ± 25.3	83.9 ± 5.7	18.3–28.0	23.1 ± 3.0
Payara (*Hydrolycus armatus*)	C	6	516.2–877.3	690.5 ± 122.7	467.7–837.8	647.4 ± 126.0	93.5 ± 1.9	27.3–65.7	43.6 ± 16.0
Picalón (*Pimelodus blochii*)	O	12	135.9–377.6	301.4 ± 67.4	128.1–335.9	262.8 ± 60.7	87.3 ± 4.2	18.3–27.0	21.8 ± 3.0
Piraña negra (*Serrasalmus rhombeus*)	O	8	232.0–689.3	423.6 ± 138.0	186.3–642.8	370.4 ± 134.4	86.7 ± 4.2	8.5–40.3	24.2 ± 10.7
Puño o Caribe (*Serrasalmus gouldingi*)	C	7	192.2–2060.9	659.7 ± 677.7	164.4–1929.2	602.5 ± 639.1	89.3 ± 2.8	6.8–45.5	27.4 ± 16.4
Sabaleta (*Brycon* sp.)	O	11	105.7–319.4	213.4 ± 79.2	81.6–288.3	184.3 ± 76.4	84.8 ± 4.9	25.3–30.5	28.3 ± 1.7
Simi o Mota (*Calophysus macropterus*)	C	15	851.5–2456.7	1683.3 ± 469.9	762.1–2156.0	1395.6 ± 407.0	83.0 ± 5.0	35.8–63.7	49.8 ± 7.8
Tucunaré (*Cichla ocellaris*)	C	17	85.7–756.3	466.9 ± 220.4	67.8–713.0	421.9 ± 207.4	89.2 ± 4.2	22.3–42.7	33.7 ± 6.6
Yacundá (*Crenicichla* sp.)	C	8	248.8–453.6	316.8 ± 72.4	218.6–407.7	288.0 ± 64.9	91.0 ± 2.0	18.4–27.6	22.4 ± 3.5
Total		196	8.4–2524.7	542.8 ± 576.1	6.6–2411.7	485.2 ± 516.8	87.8 ± 5.4	6.8–65.7	34.7 ± 13.8

FH: feeding habit (C = carnivorous; O = omnivorous).

**Table 3:** Sociodemographic variables, estimated weekly mercury intake and mercury concentrations in human matrices.

Variable	Mean ± SD	Median	IQR
Age (years)	36.9 ± 14.3	36.0	20.0
Stature (cm)	152.8 ± 57.1	152	10
Weight (kg)	57.1 ± 10.2	57	12.5
Residence time (months)	181.1 ± 152.6	156.0	144.0
Blood Hg (µg/L)	80.4 ± 43.9	69.3	37.8
Urine Hg (µg/L)	15.1 ± 11.4	11.7	11.6
Hair Hg (µg/g)	19.8 ± 10.1	17.1	8.6
Hair MeHg (µg/g)	16.8 ± 8.6	14.6	7.7
Hg Weekly intake (µg/kg/week)	32.7 ± 21.4	37.0	25.7
MeHg Weekly intake (µg/kg/week)	29.0 ± 19.6	24.4	23.5
Hair MeHg/Pregnant (µg/g) (*n* = 13)	17.2 ± 8.0	14.3	13.1
MeHg Weekly intake/Pregnant (µg/kg/week)	45.3 ± 29.8	32.8	31.8

**Table 4:** Sociodemographic and nutritional factors associated with hair mercury concentrations (µg/g).

Variable	Mean ± SD	Median	IQR	*p*
Education: Illiterate	15.9 ± 7.2	15.3	8.4	0.023
Education: Primary	19.7 ± 9.9	17.0	6.5	
Education: Secondary	21.8 ± 12.2	18.2	13.6	
Residential history: Same area	14.9 ± 5.2	15.0	5.2	<0.001
Residential history: Different zones	24.5 ± 13.6	20.0	13.1	
Fish source: Apaporis River	21.5 ± 10.6	18.7	11.2	0.001
Fish source: Apaporis and tributaries	17.7 ± 9.7	16.0	5.7	
Fish source: Other rivers	16.0 ± 8.1	15.3	14.0	

**Table 5:** Spearman correlations between hair mercury and other variables.

Variable	ρ	*p*
Age	0.128	0.048
Residence time	−0.212	0.001
Hair MeHg	0.790	<0.001
Blood Hg	0.794	<0.001
Urinary Hg	0.364	<0.001

**Table 6:** Multivariate model of factors associated with hair mercury concentrations.

Variable	ρ	t	*p*
Sex (female)	−0.063	−1.390	0.166
Fish source: streams/other rivers	−0.090	−1.979	0.049
Age	−0.120	−2.666	0.008
Blood Hg (µg/L)	0.707	14.187	0.000
Urine Hg (µg/L)	0.102	2.057	0.041

## Data Availability

The data presented in this study are available on request from the corresponding author. The data are not publicly available due to ethical restrictions related to the protection of indigenous communities.

## Abbreviations

The following abbreviations are used in this manuscript:

Hg	Mercury
MeHg	Methylmercury
ASGM	Artisanal and Small-Scale Gold Mining
ACITAVA	Association of Indigenous Captains of Taraira Vaupés
WHO	World Health Organization
DMA	Direct Mercury Analyzer
